# Distinct Cell-Cycle Control in Two Different States of Mouse Pluripotency

**DOI:** 10.1016/j.stem.2017.09.004

**Published:** 2017-10-05

**Authors:** Menno ter Huurne, James Chappell, Stephen Dalton, Hendrik G. Stunnenberg

**Affiliations:** 1 Department of Molecular Biology, Faculty of Science, Radboud University, 6525GA Nijmegen, the Netherlands; 2 Paul D. Coverdell Center for Biomedical and Health Sciences, Department of Biochemistry and Molecular Biology, The University of Georgia, Athens, GA 30602, USA

**Keywords:** embryonic stem cells, pluripotency, cell cycle, G1 checkpoint, retinoblastoma protein, ERK-signaling, diapause

## Abstract

Mouse embryonic stem cells (ESCs) cultured in serum are characterized by hyper-phosphorylated RB protein, lack of G1 control, and rapid progression through the cell cycle. Here, we show that ESCs grown in the presence of two small-molecule inhibitors (2i ESCs) have a longer G1-phase with hypo-phosphorylated RB, implying that they have a functional G1 checkpoint. Deletion of RB, P107, and P130 in 2i ESCs results in a G1-phase similar to that of serum ESCs. Inhibition of the ERK signaling pathway in serum ESCs results in the appearance of hypo-phosphorylated RB and the reinstatement of a G1 checkpoint. In addition, induction of a dormant state by the inhibition of MYC, resembling diapause, requires the presence of the RB family proteins. Collectively, our data show that RB-dependent G1 restriction point signaling is active in mouse ESCs grown in 2i but abrogated in serum by ERK-dependent phosphorylation.

## Highlights

• 2i ESCs have a longer G1-phase and a shorter G2-phase than serum-grown cells
• RB family proteins control the restriction point and elongate the G1-phase in 2i ESCs
• ERK pathway signaling abrogates the restriction point in serum-grown ESCs
• Diapause-like quiescence of ESCs also depends on RB-mediated G1 regulation

## Graphical Abstract

**Figure:**
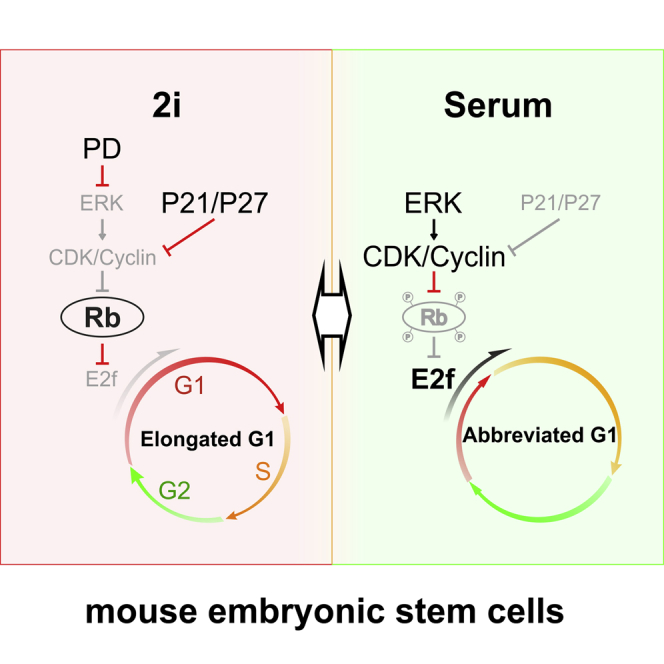

## Teaser

It is widely thought that embryonic stem cells have an unusual and very rapid cell cycle, lacking normal G1 regulation. Stunnenberg and colleagues show that, in mouse ESCs, G1 regulation is, in fact, intact but abrogated in serum by ERK signaling and RB phosphorylation.

## Introduction

One characteristic of embryonic stem cells (ESCs) is that they proliferate at an unusually rapid pace, with a doubling time of ∼12–14 hr. The highly proliferative nature of pluripotent ESCs is attributed to the lack of G1-phase control, a feature that has been intimately linked to pluripotency (Singh and Dalton, 2009). Constitutively active kinases and the absence of CDK-inhibitor proteins ensure rapid progression into S-phase, resulting in an extremely short G1-phase. This model is, however, based on studies performed in ESCs grown in serum (hereinafter termed serum ESCs). Recent studies have indicated that ESCs cultured in defined medium in the presence of two small-molecule inhibitors, PD0325901 and CHIR99021, (hereinafter termed 2i ESCs) more closely reflect pluripotent ESCs from the inner cell mass (often referred to as ground-state), whereas ESCs cultured in serum are more similar to pluripotent cells from post-implantation embryonic stages (Marks et al., 2012, Nichols and Smith, 2009, Weinberger et al., 2016). Our analysis of the cell cycle in 2i ESCs indicates that G1 control in ground-state pluripotent ESCs is distinct from that in serum ESCs.

## Results

**Figure 1:**
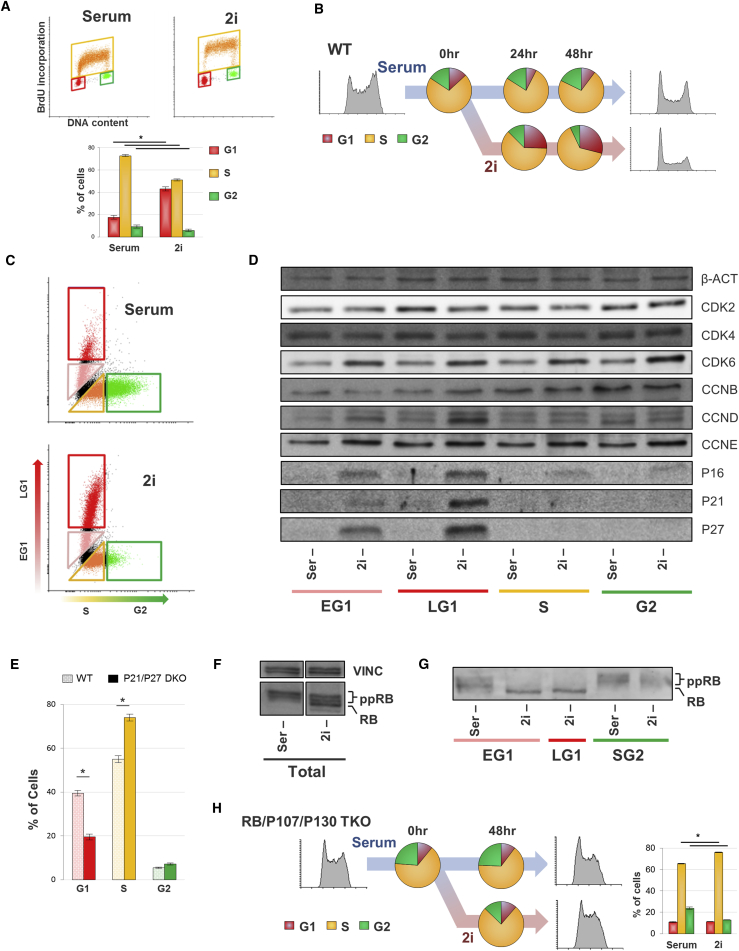
G1-Phase Is Elongated upon Adaptation of ESCs to 2i Conditions For a Figure360 author presentation of Figure 1, see the figure legend at http://dx.doi.org/10.1016/j.stem.2017.09.004. (A) DNA staining using PI in combination with BrdU incorporation shows a higher number of 2i R1 ESCs residing in G1 phase when compared to serum R1 ESCs. Significance was tested using the two-tailed Student’s t test; ^∗^p < 0.05. (B) Cell-cycle analysis using BrdU/PI shows that the rapid increase in the number of cells residing in G1-phase upon transition from serum to 2i occurs within 48 hr. Similar results were observed in several different male and female ESC lines. (C) FUCCI reporter expression shows that 2i ESCs reside longer in G1 phase, whereas the combined S-/G2-phase is shortened in 2i ESCs compared to serum ESCs. Indicated are the gates as used for sorting. (D) Western blot analysis of cell-cycle proteins involved in progression into S-phase in serum and 2i ESCs showing specific upregulation of the CDK inhibitors in 2i ESCs during both early and late G1-phase (EG1 and LG1, respectively). Three independent experiments showed similar results. (E) Distribution of cells over different phases of the cell cycle as determined by BrdU/PI staining performed in triplicate, using three independent P21/P27 DKO clones. A two-tailed Student’s t test was used to determine significance; ^∗^p < 0.05. (F) Western blot analysis of total cell populations showing only phosphorylated RB in serum ESCs. Western blot is representative of three independent experiments. (G) As in (C), using sorted early- and late-G1-phase as well as S-/G2-phase of ESCs, showing hypo-phosphorylated RB in G1-phase and phosphorylated RB (phospho-RB) in S-/G2-phase in 2i ESCs. At least two independent experiments showed similar results. (H) Cell-cycle analysis on RB/P107/P130 TKO ESCs using BrdU in combination with PI, showing an unaltered number of cells in G1-phase upon adaptation from serum to 2i. A decrease in the number of cells in G2-phase was observed. Results are representative of at least two independent experiments (^∗^p < 0.05, two-tailed Student’s t test). All values and error bars represent the mean ± SD. See also Figures S1 and S2 and Table S1. Figure360: An Author Presentation of Figure 1

To assess the global effect of the 2i culture conditions on the mouse ESC cell cycle, we used bromodeoxyuridine (BrdU) incorporation and propidium iodide (PI) staining in combination with flow cytometry to determine the distribution of cells over the different phases of the cell cycle. In line with literature, the majority of serum ESCs reside in the S-phase, whereas the rest are roughly equally distributed between the G1 phase and G2 phase (Figure 1A). Conversely, the number of 2i ESCs in S-phase is reduced, whereas the number of cells in the G1-phase is strongly increased, which is largely effectuated already within 24 hr of adaptation from serum to 2i conditions, or vice versa (Figures 1B and S1A). The adaptation of serum ESCs to 2i conditions does not affect the expression of key pluripotency factors (Marks et al., 2012), indicating that these changes in cell cycle are not the result of differentiation (Figure S1B). Similar results were obtained in multiple karyotyped wild-type (WT) ESC lines, both male and female with several different genetic backgrounds, and in induced pluripotent stem cells (iPSCs) (Li et al., 2009a) (Figures S1C and S1D; Table S1).

Serum-grown ESCs have been reported to contain Nanog-high and Nanog-low subpopulations, with the former exhibiting a higher degree of pluripotency (Chambers et al., 2007, Kalmar et al., 2009). Both populations display a serum-type cell-cycle distribution (Figure S1E), indicating that the elongated G1-phase is characteristic of ground-state 2i ESCs.

To gain further insight, we generated mouse ESCs expressing the FUCCI reporters (Sakaue-Sawano et al., 2008). The FUCCI system enables fluorescence-activated cell sorting (FACS) of cells from different phases of the cell cycle and, importantly, determination of the length of the different phases, because the reporter activity increases proportionally to the time spent in a specific phase (Figure S1F). The analysis confirms that 2i ESCs have a markedly longer G1-phase and shorter S-/G2-phases, relative to serum ESCs (Figures 1C and S1G). As serum and 2i ESCs proliferate at a roughly similar pace (Figure S1H) (Tamm et al., 2013), these changes appear to have a compensatory effect on the length of the cell cycle.

The abbreviated G1-phase in serum ESCs is accompanied by the accelerated expression of histones during G1-/S-phase transition (Medina et al., 2012). To assess whether serum and 2i ESCs differ in this respect, we performed RNA sequencing (RNA-seq) on 2i and serum ESCs in G1-phase. Gene ontology (GO)-term analysis on differentially expressed genes (Figure S1I; fold change > 2, p < 0.05) revealed higher expression of genes associated with S-phase entry and chromatin assembly in serum ESCs (Figure S1J). Replication-dependent histone genes are more highly expressed in serum ESCs, when compared to 2i ESCs, both in late G1-phase and in early G1-phase (Figure S1K; data not shown). The accelerated progression through G1-phase, upregulation of histone gene expression, enrichment of the E2F binding motif at highly expressed genes (Figure S1L), and increased expression of the E2F family members and known E2F target genes (Figure S1M) suggest elevated activity of the RB-E2F pathway in serum ESCs. E2F1 chromatin immunoprecipitation sequencing (ChIP-seq) revealed that ∼5% of the binding sites displayed differential binding (2-fold change, p < 0.05) between serum and 2i (Figure S1N). However, E2F1 occupancy did not correlate with differential RNA expression (R^2^ = 0.08). Thus, differential E2F binding is very unlikely to drive differential gene expression in serum and 2i.

To assess whether cyclin/CDK activity upstream of the RB-E2F pathway explains the distinct cell-cycle patterns, we determined the expression of key regulators of cell-cycle progression through G1-phase. Quantitation of their protein levels throughout the cell cycle revealed that CDK2 and CDK4 are slightly lower, whereas CDK6, CCND, and CCNE are slightly higher expressed in 2i ESCs (Figure 1D). The most notable difference, however, is the higher protein levels of the CDK inhibitors: P16, P21, and P27 are nearly exclusively expressed only in 2i ESCs and primarily during the late G1-phase (Figure 1D). Importantly, differential expression of CDK-inhibitors in 2i and serum ESCs parallels their higher RNA levels in inner cell mass as compared to the epiblast (Boroviak et al., 2015) (Figure S2A).

Deletion of P21 or P27 alone did not significantly alter the cell cycle; however, the combined deletion of P21 and P27 resulted in a decrease in the number of cells in G1-phase as well as lowered expression of the FUCCI reporters. The deletion of P16, alone or in combination with P21, did not significantly affect the number of cells in G1-phase (data not shown). These results indicate that the CDK-inhibitor proteins have partially overlapping functions and that the length of G1-phase in 2i ESCs is controlled by the sum of P21/P27 inhibitory action (Figures 1E and S2B–S2D).

To assess the net effect of differential CDK activity between serum and 2i ESCs, we determined the expression and phosphorylation status of their downstream targets, the RB family proteins RB, P107, and P130. In the hypo-phosphorylated form, these proteins bind to and inhibit the activity of DNA-bound E2F transcription factors, thereby slowing down cell-cycle progression during G1-phase. Upon successive phosphorylation of RB, its interaction with E2F transcription factors is lost, leading to the activation of genes involved in progression into S-phase (Dick and Rubin, 2013, Hirschi et al., 2010). Western blot analysis shows that hyper-phosphorylated forms of RB are detected in serum ESCs, whereas RB appears hyper- as well as hypo-phosphorylated in 2i FUCCI ESCs, which was confirmed by phosphatase treatment (Figures 1F and S2E). Strikingly, in both the early and late G1 phases of 2i FUCCI ESCs, only hypo-phosporylated RB is detected (Figure 1G). These results were confirmed in G1-phase sorted Suv39h WT ESCs (Lehnertz et al., 2003) and in E14 ESCs (Figure S2F; data not shown). Phosphorylated RB is, however, present in S-/G2-phase in both 2i and serum ESCs (Figures 1G and S2F). In P21/P27 double knock out (DKO) ESCs cultured in 2i, the hypo-phosphorylated form of RB is hardly detected. This indicates that the expression of CDK inhibitors in 2i prevents phosphorylation of RB (Figure S2G). Besides hypo-phosphorylated RB, P107 protein levels are higher in 2i ESCs, which could aid in reinstating the G1 checkpoint (Figure S2H). Together, these results suggest that E2F activity is higher in serum and that E2F activity is inhibited by hypo-phosphorylated RB family proteins in 2i.

We used RB knockout (KO) and RB/P107/P130 triple-knockout (TKO) ESCs (Dannenberg et al., 2000) to corroborate and extend our hypothesis that the elongated G1-phase in 2i ESCs is mediated by the RB family proteins. In TKO ESCs, the number of cells in G1-phase remains the same when shifting from serum to 2i conditions, indicating that the RB family proteins are essential for elongation of G1-phase in 2i ESCs (Figure 1H). In 2i ESCs, the cell-cycle distribution of only RB KO cells is changed, but to a lesser extent than in 2i TKO ESCs (Figure S2I). Note that the expression of key pluripotency genes and colony formation are not affected in TKO ESCs (Figures S2J and S2K). Taken together, the RB family proteins are involved in the control of the G1-phase in 2i ESCs.

**Figure 2:**
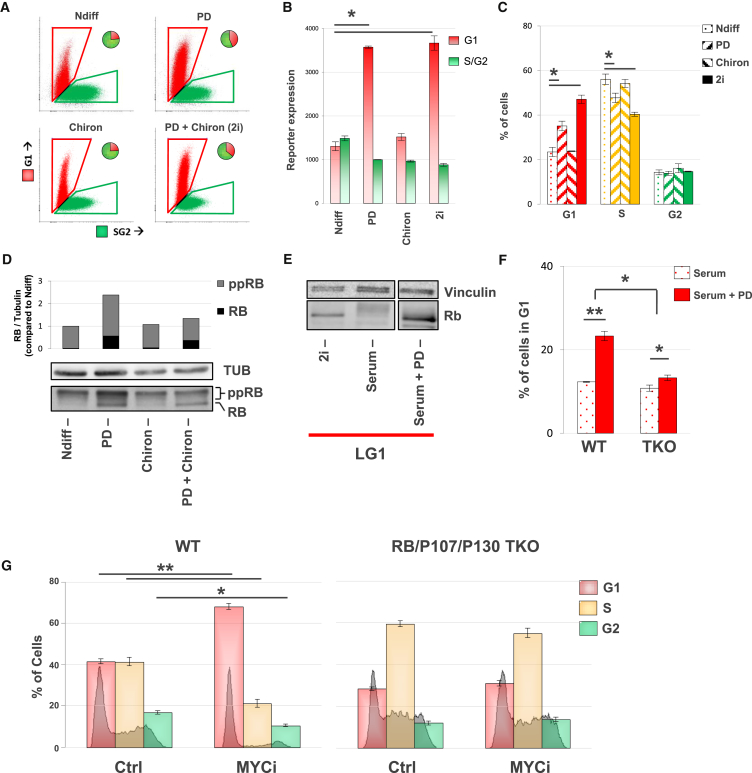
Role of ERK Signaling and RB Family Proteins in Cell-Cycle Regulation in Ground-State ESCs (A) Quantification of G1- and SG2-phases in FUCCI cells in Ndiff medium supplemented with LIF and either PD, Chiron, or both inhibitors. (B) FUCCI reporter expression shows that inhibition of the ERK signaling pathway by PD results in elongation of G1-phase. Significance was assessed by a two-tailed Student’s t test, ^∗^p < 0.05. At least two independent experiments showed these results. (C) Cell-cycle analysis using PI shows that the addition of PD results in an increase of cells in G1-phase, whereas Chiron had no significant effect (^∗^p < 0.05, Student’s t test). (D) ESCs cultured in serum-free NDiff medium + LIF supplemented with either one or both inhibitors show the presence of hypo-phosphorylated RB upon inhibition of the ERK signaling pathway by PD and not by Chiron. (E) Exposure of serum ESCs to PD results in hypo-phosphorylated and increased expression of RB. Similar results were observed in two independent experiments. (F) Quantitation of the number of cells in G1-phase of WT serum ESCs and RB/P107/P130 triple-knockout serum ESCs incubated in the presence of PD, showing that elongation of G1-phase required the RB family proteins. Bar graphs indicate mean ± SD. Comparison was performed by two-tailed Student’s t test; ^∗^p < 0.01; ^∗∗^p < 0.001; n = 3. (G) Inhibition of MYC by a small-molecule inhibitor, 10058-F4, results in a near-complete block of WT ESCs but not of RB/P107/P130 triple-knockout ESCs cultured in 2i. Error bars represent means ± SD from triplicates, representative of two independent experiments. Significance was assessed by two-tailed Student’s t test; ^∗^p < 0.001; ^∗∗^p < 0.0001. The bar charts represent the means ± SD. See also Figure S2.

To determine which signaling pathway regulates the phosphorylation status of RB, ESCs were cultured in medium + LIF supplemented either with PD0325901 (“PD”), CHIR99021 (“Chiron”), or both (“2i”). When taken out of 2i conditions, ESCs do proliferate for at least 1–2 weeks in Ndiff 227 media (Takara, formerly Ndiff N2B27, “StemCells”) + LIF. FUCCI reporter assays and PI FACS analysis indicate that the addition of PD, which blocks the ERK-signaling pathway, drives the elongation of G1-phase, whereas the GSK3 inhibitor Chiron has no effect (Figures 2A–2C). Western blot analysis detected hypo-phosphorylated RB only in ESCs cultured in the presence of PD (Figure 2D). Similarly, PD addition to serum ESCs gives rise to elevated, mostly hypo-phosphorylated RB protein (Figures 2E and S2F), possibly because of lowered cyclin D levels (Figure S2L). These effects are accompanied by an increased number of cells in the G1-phase in wild-type ESCs (Figures 2F and S2M), which is strongly reduced in TKO ESCs (Figure 2F). Chiron had, again, only a marginal effect on the length of G1 phase in serum ESCs, as in serum-free conditions (Figures 2B and S2M). We conclude that pluripotent 2i ESCs do possess an active G1 restriction point that, in serum ESCs, is abrogated by ERK signaling and RB hyper-phosphorylation.

It has recently been shown that inhibition of C-MYC and N-MYC in 2i ESCs results in quiescence, mimicking in vivo diapause (Scognamiglio et al., 2016). To test whether the RB family proteins are required for G1-arrest, we treated WT and RB/P107/P130 TKO ESCs with the MYC inhibitor (MYCi). Upon MYC inhibition, WT 2i ESCs stall in the G1-phase, in line with a recent report (Scognamiglio et al., 2016). In RB/P107/P130 TKO ESCs, however, the number of cells in G1-phase did not significantly increase upon MYC inhibition (Figure 2G). We conclude that the RB/P107/P130-mediated restriction point is required to stall the cells in G1 in a dormancy-like state.

## Discussion

It has been postulated that an abbreviated G1-phase is characteristic of ESCs and ensures maintenance of pluripotency. Our study shows that elongation of the G1-phase in 2i ESCs is the most prominent difference in the cell cycle when compared to serum ESCs, yet 2i ESCs are highly pluripotent and perform even better in chimera formation then serum ESCs (Alexandrova et al., 2016). Together with the observations that 2i ESCs have a lower propensity to differentiate (Marks et al., 2012, Ying et al., 2008), our findings imply that a short G1-phase is not an intrinsic property of pluripotent ESCs. Our data corroborate and significantly extend previous observations in serum ESCs (Gonzales et al., 2015, Li et al., 2012) by revealing the mechanism of the fundamentally different G1 control in 2i versus serum ESCs.

The fibroblast growth factor (FGF)/ERK signaling plays an important role during early embryonic development, and loss of FGF4 results in impaired cell proliferation of inner cell mass (ICM) cells (reviewed in Dorey and Amaya, 2010, Lanner and Rossant, 2010). Our results indicate that signaling via the ERK pathway results in a decrease in hypo-phosphorylated RB, loss of the restriction point, and an abbreviated G1-phase in serum ESCs. As the FGF/ERK pathway plays a crucial role in early fate specification during the implantation stage (Lanner and Rossant, 2010) that coincides with bursts of cell proliferation (Snow, 1977), we lend further support to the model in which 2i ESCs resemble naive pluripotent (ICM) cells by showing that FGF/ERK signaling results in the shortening of G1-phase in epiblast (serum) ESCs.

Besides the difference in G1-phase between serum and 2i ESCs, a shortening of S/G2-phase was observed as well. These observations are in concordance with marked changes in cell cycle during early embryonic development (reviewed in Bouldin and Kimelman, 2014, Duronio et al., 1996). It is tempting to speculate that this is caused by observed discrepancies in the expression of cell-cycle regulators, such as differences in cyclin E expression during S-phase and the higher expression of all Cdc25 isoforms during G2-phase (unpublished data) (Bouldin and Kimelman, 2014, Farrell et al., 2012, Sansam et al., 2015). Moreover, differences in epigenetic make-up and chromatin landscape between serum and 2i ESCs (Habibi et al., 2013, Marks and Stunnenberg, 2014) could possibly affect the length of these phases as well (Shermoen et al., 2010).

Collectively, our results provide a paradigm shift in how the cell cycle is regulated in pluripotent stem cells. Surprisingly, 2i ESCs do harbor an intact G1 control, which is lost upon adaptation to serum conditions due to increased ERK signaling and RB hyper-phosphorylation. Our data imply a revised conceptual framework for cell-cycle regulation in pluripotent ESCs and during early embryonic development.

## STAR★Methods

### Key Resources Table

**Table:** 

REAGENT or RESOURCE	SOURCE	IDENTIFIER
**Antibodies**		

Goat polyclonal anti-bActin	Abcam	Cat#ab8229; RRID: AB_306374
Rabbit polyclonal anti-CyclinB1	Santa Cruz	Cat#sc-752; RRID: AB_2072134
Polyclonal Goat anti-CyclinD1/2	R&D systems	Cat#AF4196; RRID: AB_2070559
Rabbit polyclonal anti-CyclinE	Santa Cruz	Cat#sc-481; RRID: AB_2275345
Rabbit polyclonal anti-CDK2	Santa Cruz	Cat#sc-163; RRID: AB_631215
Rabbit polyclonal anti-DK4	Santa Cruz	Cat#sc-260; RRID: AB_631219
Rabbit polyclonal anti-CDK6	Santa Cruz	Cat#sc-177; RRID: AB_631225
Mouse monoclonal anti-E2F1	Merck Millipore	Cat#05-379; RRID: AB_2096772
Rabbit polyclonal anti-P16	Santa Cruz	Cat#sc-1207; AB_632106
Mouse monoclonal anti-P21	Santa Cruz	Cat#sc-6246; RRID: AB_628073
Rabbit polyclonal anti-P27	Santa Cruz	Cat#sc-528; RRID: AB_632129
Mouse monoclonal anti-RetinoBlastoma	BD PharMingen	Cat#554136; RRID: AB_395259
Rabbit polyclonal anti-Vinculin	Santa Cruz	Cat#sc-5573; RRID: AB_2214507
Mouse anti-BrdU-FITC	Biolegend	Cat#364104; RRID: AB_2564481

**Chemicals, Peptides, and Recombinant Proteins**		

Ndiff 227	Takara	Cat#Y40002
CHIR99021	Axon	Cat#1386
PD0325901	Axon	Cat#1408
LIF	Millipore	Cat#ESG1107
cOmplete	Roche	Cat#04693132001
PhosSTOP	Roche	Cat#04906845001
Propidium Iodide	Sigma	Cat#P4170
5-Bromo-2-deoxyuridine	Sigma	Cat#B5002
Hoechst 33342	Invitrogen	Cat#H1399

**Critical Commercial Assays**		

Ribo-Zero Gold Kit	Epicenter	Cat#MRZG126
TruSeq RNA Sample Prep Kit	Illumina	Cat#RS-122-2001
Wizard Genomic DNA extraction kit	Promega	Cat#A1120
Lipofectamine-LTX	ThermoFisher Scientific	Cat#15338100
Platinum Pfx Polymerase	Invitrogen	Cat#11708013
PCR Purification Kit	QIAGEN	Cat#28106
DNA Ligation Kit, Mighty Mix	Takara	Cat#6023
NextFlex-96 DNA Barcodes	Bioo Scientific	Cat#NOVA-514106

**Deposited Data**		

RNA-seq	This paper	GEO: GSE85690
ChIP-seq	This paper	GEO: GSE85690
RNA-seq	(Boroviak et al., 2015)	E-MTAB-2958
Unprocessed data	This paper	http://dx.doi.org/10.17632/9hcdttwzyb.1

**Experimental Models: Cell Lines**		

E14	ATCC	Cat#CRL-1821; RRID:CVCL_9108
R1	ATCC	Cat#SCRC-1011; RRID:CVCL_2167
EB5	Laboratory of Hitoshi Niwa	N/A
SV8	(ten Berge et al., 2011)	N/A
SV7	(ten Berge et al., 2011)	N/A
TNGA	(Chambers et al., 2007)	N/A
XT67E1	(Penny et al., 1996)	N/A
Suv39h WT	(Lehnertz et al., 2003)	N/A
ES_Tsix-stop	(Luikenhuis et al., 2001)	N/A
iPS WT	(Li et al., 2009b)	N/A

**Oligonucleotides**		

gRNA_Cdkn1a-01_Fwd: CACCGTTGTCTCTTCGGTCCCG	This paper	N/A
gRNA_Cdkn1a-01_Rev: AAACCGGGACCGAAGAGACAAC	This paper	N/A
gRNA_Cdkn1a-02_Fwd: CACCGTCCGACCTGTTCCGCAC	This paper	N/A
gRNA_Cdkn1a-02_Rev: AAACGTGCGGAACAGGTCGGAC	This paper	N/A
gRNA_Cdkn1b_Fwd: CACCGCGGATGGACGCCAGACAAG	This paper	N/A
gRNA_Cdkn1b_Rev: AAACCTTGTCTGGCGTCCATCCGC	This paper	N/A

**Recombinant DNA**		

pSpCas9(BB)-2A-GFP (PX458)	(Ran et al., 2013)	Addgene: 48138
pcDNA3	From Atsushi Miyawaki Lab	N/A
pCAG-IRES-PURO	From Austin Smith Lab	N/A
pCAG-IRES-NEO	From Austin Smith Lab	N/A

**Software and Algorithms**		

Flowing Software		http://flowingsoftware.com/
BWA	(Li et al., 2009b)	http://bio-bwa.sourceforge.net/
Bowtie	(Langmead et al., 2009)	http://bowtie-bio.sourceforge.net/
MMSeq	(Turro et al., 2011)	https://github.com/eturro/mmseq
Picard		http://broadinstitute.github.io/picard
Macs2	(Zhang et al., 2008)	https://github.com/taoliu/MACS/tree/master/MACS2
DESeq2	(Love et al., 2014)	http://bioconductor.org/packages/release/bioc/html/DESeq2.html
Homer	(Heinz et al., 2010)	http://homer.ucsd.edu/homer/motif/
DAVID	(Huang et al., 2009)	https://david.abcc.ncifcrf.gov/

### Contact for Reagent and Resource Sharing

Further information and requests for resources and reagents should be directed to and will be fulfilled by the Lead Contact, Henk Stunnenberg (h.stunnenberg@ncmls.ru.nl).

### Experimental Model and Subject Details

#### Cell lines and culture conditions

All ESC lines, described including sex in Table S1, were cultured in either serum medium, containing 15% fetal calf serum (Hyclone), penicillin/streptomycin, sodium pyruvate, 0.1mM 2-mercaptoethanol, and 1000 U/mL LIF or Ndiff 227 medium (Takara, formerly Ndiff B2N27 – “StemCells”), supplemented with CHIR99021 at 3 mM(Axon), PD0325901 at 1mM(Axon) and 1000 U/mL LIF (Millipore) (“2i”) in a 37°C humidified incubator with 5% CO_2_. Prior to transition from serum medium to 2i medium or vice versa cells were washed twice with PBS.

### Method Details

#### Establishment of R1 FUCCI ESCs

mKO2-CDT1 (30-120) and AZ1-GEMININ (1-110) gene fragments were PCR amplified from 100 ng pcDNA3 backbone (gift from Miyawaki and colleagues (Sakaue-Sawano et al., 2008)) using modified T7 and SP6 primers with EcoR1 restriction sites at their 5′ ends. PCR amplification was performed using Platinum Pfx DNA polymerase (Invitrogen) according to manufacturer protocol. After purification using the PCR purification Kit (QIAGEN) these amplicons were digested with EcoR1 (NEB) according to manufacturer guidelines for 8 hr at 37°C. In addition, 5 μg expression vector containing the pCAG promoter and either the puromycin or the neomycin selection marker (a gift from Austin Smith and colleagues), was digested and treated with calf intestinal phosphatase (NEB). Next, the inserts and the expression plasmids were verified and purified by gel electrophoresis. The mKO2-CDT1 (30-110) fragment was ligated into pCAG-IRES-PURO and the AZ1-GEMININ (1-110) into pCAG-IRES-NEO expression plasmid overnight at 16°C using the DNA Ligation Mighty Mix Kit (Takara). The ligation products were transformed into DH5α E. Coli and plated onto agar plates containing carbenicillin (100 μg/mL). Next day colonies were picked and DNA was sent for sequencing. Clones of interest were amplified in LB containing carbenicillin (100 μg/mL) and DNA was isolated using the HP Endotoxin-free Maxi Prep kit (Sigma). For transfection, we used a two-step transfection selection strategy. First, pCAG-CDT1-PURO was transfected in mouse R1 ESCs and selected with puromycin (1 μg/ml) for approximately 2 weeks, and stable cell lines were isolated. Next, pCAG-GEMININ-NEO was transfected into the cell lines, and selected with G418 (200 μg/ml) for 2 weeks, and stable lines were isolated.

#### Western Blot

Cells were lysed in RIPA buffer with fresh cOmplete, EDTA-free protease inhibitor Cocktail (Roche) and PhosSTOP (Roche). Cell extracts were separated by SDS– PAGE and then transferred to nitrocellulose membranes in 20 mM Tris- HCl [pH 8.0], 150mM glycine, 20% (v/v)d methanol. Membranes were blocked with 5% (w/v) nonfat dry milk in Tris-buffered saline with Tween 20 (TBST; 20 mM Tris-HCl [pH 7.6], 0.1% Tween 20, 137 mM NaCl), incubated with primary antibodies, then secondary antibodies, and detected with ECL reagents (Amersham Biosciences).

#### Flow cytometry

The BD FACS Aria cell sorter was used to analyze and sort FUCCi ESCs. For cell cycle analysis cells were pulsed with 20 μM BrdU for 30 min, harvested by trypsinisation and fixed over night in 70% ethanol at 4°C. After denaturation of the DNA using 2N HCl + 0.5% Triton X-100 for 30 min at room temperature, neutralization with 0.1M Na_2_B_4_O_7_ (pH 8.5), samples were incubated with the anti-BrdU antibody over night at 4°C. Next day, the samples were stained with Propidium Iodide staining solution (10 ug/ml PI [Sigma, P4170] and 0.2mg/mL RNase A in PBS) over night at 4°C. Samples of at least 10000 cells were acquired using a FACScalibur flow cytometre (Becton Dickinson). Subsequent analysis was done with Flowing Software. For the cell cycle analysis of Nanog-GFP ESCs cells were incubated with Hoechst 33342 (Invitrogen) for one hour at 37°C and analyzed on the BD FACS Aria.

#### Colony formation assay

Serum grown ESCs were seeded at a dilution of one cell per well into 96-well plates using the BD FACS Aria, as described in (Fedr et al., 2013), containing either serum or 2i medium. The cells were cultured for two weeks and the number of colonies was assessed in a blind manner.

#### ChIP-seq

Serum and 2i ESCs were fixed using 1% PFA for 10 min at room temperature. Subsequently, fixation was quenched by adding 1.25M glycine to a final concentration of 0.125M. After sonication the material of 4-5 million cells and 5 μL E2F1 antibody were used per ChIP. ChIP enrichment was assessed by qPCR and 2ng of DNA was used for library construction. Paired-end 43bp deep sequencing was performed using Illumina’s NextSeq 500 sequencer.

#### Karyotype Analysis

For karyotype analysis chromatin of 0.5 million cells was sonicated and decrosslinked over night. Next genomic DNA was purified and used for library construction. Paired-end 43bp deep sequencing was performed using Illumina’s NextSeq 500 sequencer. Reads were mapped using BWA and unique reads per chromosome were normalized by total reads.

#### RNA-seq

Cells were sorted and total RNA was extracted using Trizol (Life Technologies) according to manufacturer’s instructions. After DNase treatment, 5 μg of extracted RNA was depleted from ribosomal RNA using Ribo-Zero Gold Kit (Epicenter Madison, Winsconsin, USA). After fragmentation of the rRNA-depleted RNA, 500ng was reverse-transcribed using Super Script III Reverse Transcriptase (Invitrogen) and random primers (Invitrogen) following the manufacturer’s instructions. Next, libraries were prepared using the TruSeq RNA Sample Prep Kit (Illumina) following the manufacturer’s instructions. Libraries were indexed using NEXTflex adapters (Bioo- Scientific Corporation, Austin, TX, USA), and the quality was assessed by qPCR and Bioanalyzer (BioRad). Single-end 43bp deep sequencing was performed on Illumina instruments using TruSeq reagents (Illumina, San Diego, CA, USA), according to manufacturer’s instructions.

#### Genome editing with CRISPR-Cas9

CRISPR-Cas9 gene editing was used to knock out *cdkn1a* (P21) and *cdkn1b* (P27). In brief, gRNAs were designed using the online tool (crispr.mit.edu) and cloned into the plasmid Cas9(BB)-2A-GFP (Addgene plasmid 48138) using the Bpi1 restriction sites as described previously (Cong et al., 2013). FUCCI serum ESCs were transfected using lipofectamine-LTX (life technologies). After 48 hr, GFP+ cells were sorted with a BD FACS Aria. Cells were split at clonal density and after approximately 7 days colonies were picked for expansion. Genomic DNA from individual clones was extracted using the Wizard Genomic DNA extraction kit. The targeted region was PCR amplified and Sanger Sequenced. gRNA oligonucleotides were as follows: Cdkn1a-01_Fwd: CACCGTTGTCTCTTCGGTCCCG, Cdkn1a-01_Rev: AAACCGGGACCGAAGAGACAAC, Cdkn1a-02_Fwd: CACCGTCCGACCTGTTCCGCAC, Cdkn1a-02_Rev: AAACGTGCGGAACAGGTCGGAC Cdkn1b_Fwd: CACCGCGGATGGACGCCAGACAAG, Cdkn1b_Rev: AAACCTTGTCTGGCGTCCATCCGC.

### Quantification and Statistical Analysis

Bar charts represent the mean ± standard deviation of the mean (SD). When comparing two conditions, statistical differences were assessed in Microsoft Excel with a paired two-tailed Student’s t test unless otherwise indicated in the legends. A p value of < 0.05 was considered significant unless stated differently and the exact degree of significance as indicated by asterisks is stated in the legends. Pie charts display the means of an experiment performed in triplicate representative for at least two independent experiments. Quantification and statistics belonging to the pie charts are included in the figure or Table S1.

### Data and Software Availability

#### Software

BWA and bowtie were used for ChIP-seq and RNA-seq, respectively, to align sequencing reads to the mouse genome (mm9) using default parameters. For RNA-seq transcript quantification was performed using the MMSeq package and after setting a threshold of at least 50 reads over the gene body in either serum or 2i the DESeq2-package was used to call differentially expressed genes (log2-fold change > 1 and a p value < 0.05) (Anders and Huber, 2012). Normalized read counts were subsequently used to calculate RPKM values. For ChIP-seq picard tools was used to remove duplicates (http://broadinstitute.github.io/picard) and the Encode blacklist was used to filter out artifact regions (Dunham et al., 2012). Next, macs2 was used to call peaks in individual files and bedtools was used to intersect the peak-files of biological replicates. The peak-files of serum and 2i were merged using bedtools and reads over the genomic regions in resulting file were counted using bedtools multicov. The DESeq2-package was used to call differential peaks (log2-fold change > 1 and a p value < 0.05). GO-term analysis was performed with DAVID (http://david.abcc.ncifcrf.gov/). Homer software was utilized to find de novo enriched motifs in the promoters of differentially expressed genes using default settings.

#### Data resources

The accession number for the RNA-seq data of serum and 2i ESCs in G1-phase as well as the E2F1 ChIP-seq data reported in this paper is Gene Expression Omnibus (http://www.ncbi.nlm.nih.gov/geo/): GEO: GSE85690. The original unprocessed data are available through a Mendeley Database: http://dx.doi.org/10.17632/9hcdttwzyb.1.

## Author Contributions

Funding was obtained by H.G.S. and S.D. Experiments were designed by M.t.H. and H.G.S. and performed by M.t.H. and J.C. Results were analyzed and interpreted by M.t.H. and H.G.S. The manuscript was written by M.t.H. and H.G.S. and was read and edited by all authors.

## Supplemental Information

Document S1. Figures S1 and S2 and Table S1

Document S2. Article plus Supplemental Information
