# Supplementary material for: Distinct Cell-Cycle Control in Two Different States of Mouse Pluripotency
Source: Cell Stem Cell. 2017 Oct 5;21(4):449–455.e4. doi: 10.1016/j.stem.2017.09.004 (PMC5658514; doi:10.1016/j.stem.2017.09.004)
Supplement: Document S1. Figures S1 and S2 and Table S1 [file mmc1.pdf]

Cell Stem Cell, Volume *21*

Supplemental Information

Distinct Cell-Cycle Control in Two Different States of Mouse Pluripotency

Menno ter Huurne, James Chappell, Stephen Dalton, and Hendrik G. Stunnenberg

Supplemental Figures

Figure S1.

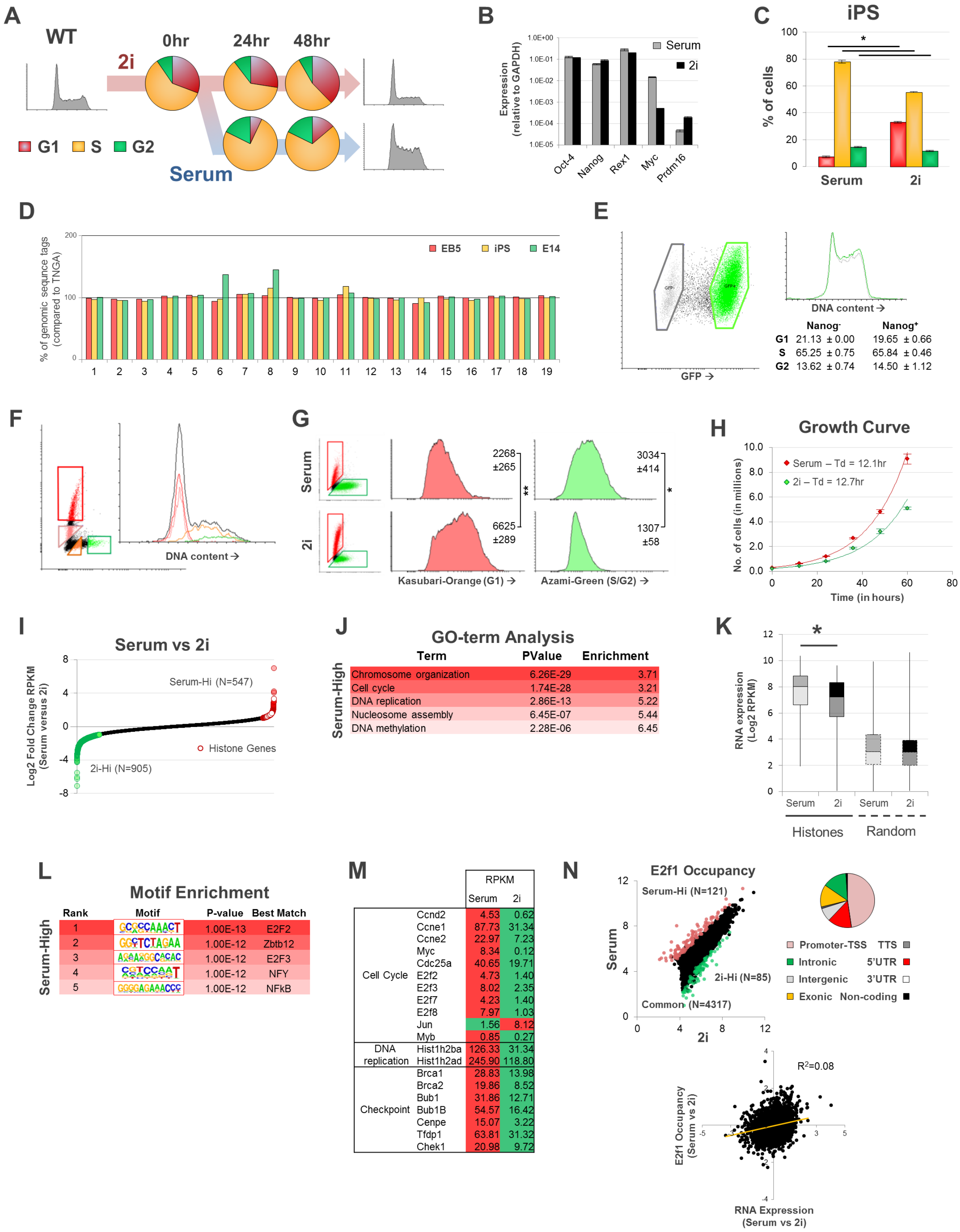

Figure S1 related to figure 1. G1-phase is elongated in 2i ESCs and is accompanied by lowered E2F activity and reduced expression of genes involved in S-phase entry

(A) Cell cycle analysis using BrdU/PI of WT 2i R1 ESCs upon adaptation to serum conditions.

(B) Expression analysis using RT-qPCR on pluripotency, serum-specific and 2i-specific genes upon adaptation of serum ESCs to 2i for 48 hours.

(C) Quantification of distribution of fully adapted serum and 2i iPS cells over the different phases of the cell cycle (Student's T-test, *p-val<0.05).

(D) Karyotype analysis (sequencing of genomic DNA) of two cell lines (EB5 and E14) and one WT iPS line. EB5 and iPS line display a normal karyotype whereas E14 shows trisomy at chromosome 6 and 8. Shown is the distribution of all sequence tags from genomic DNA of indicated cell lines over chromosomes compared to the karyotypic-normal TNG-A ESC line(Marks et al., 2012).

(E) Cell cycle analysis on $Nanog^-$ and $Nanog^+$ serum ESCs using Hoechst. Overlay histogram showing DNA content from both populations as indicated by gates in the dot plot (grey line $Nanog^-$ and green line $Nanog^+$). Numbers represent means ± standard deviations from triplicates. The experiment has been performed twice showing similar results.

(F) Determination of DNA content of 2i FUCCI ESCs using vibrant violet staining. Colored lines indicate DNA content of the cell populations sorted as indicated in dot plot.

(G) Dot plot and histogram of FUCCI reporter expression in serum and 2i ESCs. Indicated are the mean fluorescence intensities and standard deviations of triplicates. At least two independent experiments were performed showing similar results. Student's T-test, **p-val<0.001, *p-val<0.05.

(H) Growth curves and doubling times of fully adapted E14 serum and 2i ESCs. For both conditions $1.5 \cdot 10^5$ cells were seeded in triplicates and aliquots were counted at indicated times and presented as means ± SD. Two independent experiments showed similar results.

(I) Ratio of gene transcription in serum ESCs versus 2i ESCs in late G1-phase using two biological replicates.

(J) GO analysis on differentially expressed genes in serum ESCs versus 2i ESCs in late G1-phase.

(K) Boxplots of RNA expression (log2 RPKM) of histone and random chosen genes in late G1-phase showing lower expression of histone genes in 2i ESCs compared to serum. Statistical analysis was performed using the paired two tailed Student's T-test, *p-val <0.001.

(L) Motif enrichment analysis using Homer on promoter regions of genes higher expressed in serum ESCs.

(M) Expression values (RPKM) of known E2F target genes clustered based on function.

(N) ChIP-seq analysis of E2F1 occupancy is highly correlated between serum and 2i (R^2 = 0.79). The location of the E2F1 binding sites is presented as a PIE diagram: The majority of peaks is located in either the promoter-TSS or the 5'UTR and ~5% shows differential E2F1 occupancy between serum and 2i (Log2-fold change > 1, p-val <0.05). Lower panel: correlation analysis revealing absence of a correlation (R^2= 0.08) between differential E2F1 occupancy in ChIP-seq and RNA expression (RNA-seq) suggests that E2F binding does not drive the differential gene expression.

All values represent the mean ± SD.

Figure S2.

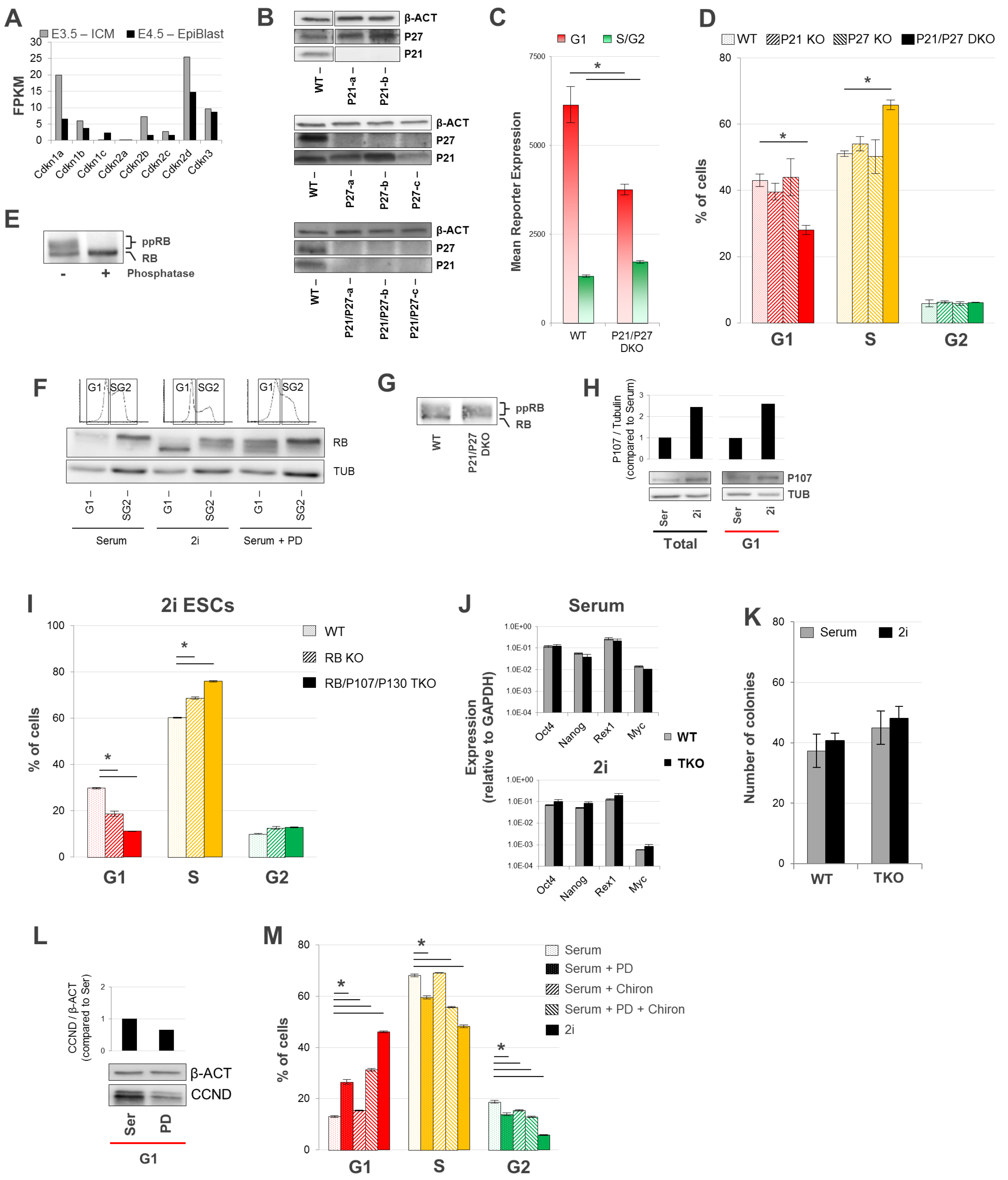

A
E3.5 – ICM
E4.5 – EpiBlast
FPKM
Cdkn1a
Cdkn1b
Cdkn1c
Cdkn2a
Cdkn2b
Cdkn2c
Cdkn2d
Cdkn3
B
β-ACT
P27
P21
WT
P21-a
P21-b
P27-a
P27-b
P27-c
P21/P27-a
P21/P27-b
P21/P27-c
C
G1
S/G2
Mean Reporter Expression
WT
P21/P27 DKO
D
WT
P21 KO
P27 KO
P21/P27 DKO
% of cells
G1
S
G2
E
ppRB
RB
Phosphatase
F
RB
TUB
Serum
2i
Serum + PD
G
ppRB
RB
WT
P21/P27 DKO
H
P107 / Tubulin (compared to Serum)
P107
TUB
Ser
2i
Total
G1
I
2i ESCs
WT
RB KO
RB/P107/P130 TKO
% of cells
J
Serum
2i
Expression (relative to GAPDH)
Oct4
Nanog
Rex1
Myc
WT
TKO
K
Serum
2i
Number of colonies
WT
TKO
L
CCND / β-ACT (compared to Ser)
β-ACT
CCND
Ser
PD
G1
M
Serum
Serum + PD
Serum + Chiron
Serum + PD + Chiron
2i
% of cells
G1
S
G2

Figure S2 related to figure 1. The elevated expression of the CDK-inhibitors P21 and P27, hypo-phosphorylation of RB and increased number of cells in G1-phase in 2i as compared to serum ESCs.

(A) RNA levels of CDK-inhibitors during early embryonic development in fragments per kilobase of exon per million fragments mapped (FPKM), adapted from (Boroviak et al., 2015) (https://doi.org/10.1016/j.devcel.2015.10.011).

(B) Western blot analysis of P21, P27 in WT, single and combined KO ESCs fully adapted to 2i.

(C) Expression of the FUCCI reporters in WT and P21/P27 DKO 2i ESCs indicates that combined knock out of P21 and P27 results in shortening of G1-phase (Student's T-test, *p-val<$0.1 \cdot 10^{-3}$).

(D) Quantitation of Flow cytometry analysis using BrdU/PI showing a significant decrease in G1-phase (Student's T-test, *p-val<0.05) of P21/P27 DKO as compared to WT 2i ESCs. No significant changes were observed in P21 KO or P27 KO 2i ESCs compared to WT 2i ESCs. Experiment performed in triplicate, using two independent P21 KO clones, three independent P27 KO clones and three independent P21/P27 DKO clones.

(E) Phosphatase treatment confirmed that the lower band present in 2i ESCs is hypo-phosphorylated RB.

(F) The Suv39h WT ESC line (Lehnertz et al., 2003) shows an elevated level of hypo-phosphorylated RB in G1-phase both in 2i as well as in serum plus PD0325901 (hereafter PD) conditions.

(G) Western blot analysis phosphorylation of RB in P21/P27 DKO 2i ESCs compared to WT 2i ESCs. Two independent clones were assessed showing similar results.

(H) Western blot analysis showing higher levels of P107 in 2i versus serum ESCs both in total cell lysates and lysates from G1-sorted cells.

(I) Cell cycle analysis using BrdU/PI on RB KO and RB/P107/P130 TKO ESCs grown in 2i. A two-tailed Student's T-test was used to assess significance of the differences (*p-val<0.05).

(J) Expression levels of pluripotency genes in RB/P107/P130 TKO ESCs cultured in 2i and serum conditions.

(K) Colony formation assay as described by (Fedr et al., 2013) performed in triplicate.

(L) Western blot analysis and its quantitation of cyclin D protein in cell lysate from G1-phase sorted cells cultured in serum plus minus the PD inhibitor.

(M) Quantitation of the distribution of serum cells upon adaptation to serum, serum plus PD, serum plus CHIR90221 (hereafter Chiron), serum plus PD and Chiron or 2i medium as assessed by BrdU/PI staining. Significance was tested using a two-tailed Student's T-test, *p-val<0.05.

Bar charts and error bars represent the mean ± SD.

Supplemental Tables

Table S1						
Sex	Cell line (strain) (number of independent experiments)	Phase	Serum		2i	
			Mean	StDev	Mean	StDev
Male	E14 (129) (n>2)	G1	12.31	0.11	29.80***	0.28
		S	69.06	1.04	60.20**	0.22
		G2	18.63	0.95	9.99**	0.24
	R1 (129) (n>2)	G1	20.61	1.06	42.08**	0.21
		S	69.60	0.84	51.90***	0.01
		G2	9.78	0.27	6.02**	0.21
	EB5 (129) (n>2)	G1	12.68	0.25	29.48***	1.29
		S	65.23	1.14	58.17***	0.81
		G2	22.08	1.30	12.34**	0.50
	SV7 (129) (n=2)	G1	8.31	0.28	13.06*	0.44
		S	76.00	0.29	73.26*	0.84
		G2	15.70	0.13	13.68*	0.39
	Suv39h WT (129) (n=1)	G1	12.54	0.39	36.82***	0.96
		S	72.44	0.41	56.51**	1.11
		G2	15.02	0.54	6.67**	0.17
	iPS (C57BL/6) (n=1)	G1	7.32	0.63	33.03***	0.73
		S	78.10	1.06	55.30**	0.33
		G2	14.58	0.63	11.68*	0.41
Female	SV8 (129) (n=2)	G1	10.33	0.79	17.45**	0.29
		S	74.63	2.06	70.72	0.38
		G2	15.04	1.27	11.83*	0.42
	TNGA (129 x C57BL/6J) (n=1)	G1	6.08	0.30	14.72**	0.88
		S	74.91	0.92	74.84	0.58
		G2	19.00	0.70	10.45**	0.34
	XT (C3H x 129) (n=2)	G1	13.86	0.43	22.48**	0.19
		S	70.29	0.24	72.26*	0.17
		G2	15.86	0.27	5.26***	0.24
	ES_Tsix-stop (129 x Cast) (n=2)	G1	12.41	0.34	27.44**	0.86
		S	70.89	0.93	66.41*	0.82
		G2	16.70	0.91	6.15**	0.41

Table S1 related to figure 1. Difference between distribution of cells over the cell cycle phases in 2i as compared to serum conditions.

Overview of serum ESC lines adapted to either serum or 2i conditions for two days. Experiments were performed in triplicate and the statistical differences between 2i and serum were calculated using the paired Student's T-test. Numbers indicate the means and standard deviations of triplicates. Indicated is the number of independent experiments showing similar results per cell line. ***p-val<0.0005, **p-val<0.005, *p-val<0.05.
